# Key informants’ perspectives on integrating community health workers into palliative care teams

**DOI:** 10.1017/cts.2024.660

**Published:** 2025-01-22

**Authors:** Taleaa Masroor, Shannon Fuller, Olivia Monton, Mahtab Vasigh, Alison P. Woods, Amn Siddiqi, Tracy B. Malone, Robert Joyner, Ronit Elk, Jill Owczarzak, Fabian M. Johnston

**Affiliations:** 1 Department of Surgery, Johns Hopkins University School of Medicine, Baltimore, MD, USA; 2 Department of Health, Behavior and Society, Johns Hopkins Bloomberg School of Public Health, Baltimore, MD, USA; 3 Department of Epidemiology, Johns Hopkins Bloomberg School of Public Health, Baltimore, MD, USA; 4 Department of Surgery, McMaster University, Hamilton, ON, Canada; 5 Department of Surgery, Beth Israel Deaconess Medical Center, Boston, MA, USA; 6 TidalHealth Richard A. Henson Research Institute, Salisbury, MD, USA; 7 University of Alabama at Birmingham Department of Medicine, Birmingham, AL, USA; 8 Division of Surgical Oncology, Department of Surgery, Johns Hopkins University School of Medicine, Baltimore, MD, USA

**Keywords:** Palliative care, community health workers, end-of-life care, patient-centered care, health disparities

## Abstract

**Introduction:**

Disparities in access to palliative care persist, particularly among underserved populations. We elicited recommendations for integrating community health workers (CHWs) into clinical care teams, by exploring perspectives on potential barriers and facilitators, ultimately aiming to facilitate equitable access to palliative care.

**Materials and Methods:**

Twenty-five stakeholders were recruited for semi-structured interviews through purposive snowball sampling at three enrollment sites in the USA. Interviews were conducted to understand perspectives on the implementation of a CHW palliative care intervention for African American patients with advanced cancer. After transcription, primary and secondary coding were conducted. Framework analysis was utilized to refine the data, clarify themes, and generate recommendations for integrating CHWs into palliative care teams.

**Results:**

Our sample comprised 25 key informants, including 6 palliative care providers, 6 oncologists, 5 cancer center leaders, 2 cancer care navigators, and 6 CHWs. Thematic analysis revealed five domains of recommendations: (1) increasing awareness and understanding of the CHW role, (2) improving communication and collaboration, (3) ensuring access to resources, (4) enhancing CHW training, and (5) ensuring leadership support for integration. Informants shared barriers, facilitators, and recommendations within each domain based on their experiences.

**Conclusion:**

Barriers to CHW integration within palliative care teams included limited awareness of the CHW role and inadequate training opportunities, alongside practical and logistical challenges. Conversely, promoting CHW engagement, providing adequate training, and ensuring support from leadership have the potential to aid integration.

## Background

Palliative care is a holistic caregiving approach aimed at improving quality of life and optimizing goal-concordant care and is recommended for patients with advanced cancer [1]. However, multilevel barriers impede its utilization, especially among African American patients [2–4]. These barriers include unequal access to care, lack of knowledge about federal, state, and local benefits, and prevalent distrust of the medical system due to historically enacted abuses and discrimination [5]. In the USA, African Americans make up approximately 12% of the overall population; however, they constitute only 8.5% of all hospice users [6]. They are also less likely to complete advanced directives and more likely to receive aggressive interventions at the end of life [6–9].

At the provider level, access challenges are exacerbated by the low number of specialty care providers and the growing number of patients requiring palliative care. This gap is projected to widen into an impossible-to-match ratio by 2030 [10]. Furthermore, access disparities worsen in resource-limited settings. Only 12% of providers in the USA practice in rural areas and retention is a challenge owing to poor infrastructure and limited career advancement [11]. Moreover, many palliative care services that are available remain underutilized, frequently due to misconceptions that conflate palliative and hospice care [12,13]. One promising way to bridge this disparity in access to palliative care is by integrating community health workers (CHWs) within oncologic and palliative care teams to provide educational and navigational support to patients who could benefit from palliative care services [14].

According to the American Public Health Association, a CHW is a “frontline public health worker who is a trusted member of and/or has an unusually close understanding of the community served”[15]. Studies on CHW-integrated healthcare delivery models among marginalized communities report improved uptake of primary and follow-up care, decreased costs, and improved health knowledge and lifestyle behaviors [16,17]. The role of CHWs in controlling disease outbreaks, delivering preventative care for chronic illnesses like diabetes and hypertension, cancer screening, and addressing psycho-emotional needs is well-documented [18–25]. As a result, many US health systems are eager to incorporate CHWs to improve the delivery of health services [26]. This growing interest is paralleled by a significant gap in the literature on the integration of CHWs within specialty care practice. As part of the preparatory phase of a multicenter randomized controlled trial, which is evaluating the effectiveness and implementation of a CHW-integrated palliative care program for African American patients with advanced cancer and their informal caregivers (NCT05407844), we conducted qualitative interviews with professional key informants including oncologists, palliative care providers, cancer center leaders, and CHWs to identify multilevel factors that may influence implementation [27]. The objective of this analysis was to characterize perspectives on the integration of CHWs into palliative care teams, including perceived barriers, facilitators, and recommendations related to integration.

## Methods

### Study design, participants, and setting

Twenty-five semi-structured professional key informant interviews were conducted between November 2022 and April 2023 across three clinical trial study sites: the Johns Hopkins Hospital (JHH) in Baltimore, MD, the University of Alabama at Birmingham Hospital (UAB) in Birmingham, AL, and TidalHealth Peninsula Regional (TH) in Salisbury, MD. Key informants were classified into four categories: (1) CHWs or other care navigators; (2) palliative and hospice care providers; (3) medical, surgical, or radiation oncology providers; or (4) cancer center leaders and administrators. The complete methodology has been previously published [28].

Purposive sampling was used to identify and recruit individuals from the three sites who had experience with similar programs or would be able to speak to the barriers and facilitators associated with implementing the intervention within their health center. Participants were identified through recommendations from each site’s principal investigator (PI) and a snowball sampling approach was employed to broaden the depth and reach of our sample. Eligible participants were approached by the research team via email. Participants who agreed to participate received a $50 gift card honorarium upon interview completion.

### Data collection

We approached 33 key informants via email after identification by site PIs, 25 of whom agreed to participate. Participants were evenly distributed across the three study sites (eight from JHH, eight from TH, and nine from UAB). We conducted interviews using a university-affiliated version of Zoom (Zoom Video Communications, Inc., San Jose, CA). All participants provided verbal informed consent. The duration of each interview fell between 60 and 90 minutes, and all interviews were audio recorded and transcribed.

A semi-structured interview guide was developed collaboratively by the PIs, interviewers, and two experienced qualitative researchers. The Interview guide was informed by the Consolidated Framework for Implementation Research to explore key informants’ perspectives on multilevel implementation factors across five main domains: the intervention, inner setting, outer setting, individuals involved, and implementation process [29]. The interviews focused on eliciting recommendations for implementing the CHW intervention within palliative care and oncology settings.

### Data analysis

As part of the larger study, we used an abductive coding process involving primary and secondary reviewers to code and organize the interview data in MAXQDA (VERBI Software, Berlin, Germany). The details of the coding process have been covered in more detail in a previous publication[28].

For this analysis, we focused on coded excerpts that included information relevant to the integration of CHWs into clinical teams and palliative care teams. We selected codes that fell within the following content areas: (1) integrating CHWs into care teams, (2) need and desire for training, (3) perceived value of CHWs, (4) perceptions of inpatient and outpatient palliative care services, and (5) training experiences. Coded data were subsequently analyzed using the framework method to facilitate thematic analysis [30,31]. As part of the framework method, three authors (TM, SF, OM) reviewed and summarized all coded segments contained within the abovementioned content areas. The lead author then developed a summary matrix to facilitate comparison across participants and domains. All analysts reviewed and discussed the summary table, and together, synthesized the data into five domains, which included perceived barriers, facilitators, and recommendations.

### Human Subjects Protections and Reporting Guidelines

This study was approved by the Johns Hopkins University School of Medicine Institutional Review Board (#00283002). It is reported using the Consolidated Criteria for Reporting Qualitative Research (COREQ) guidelines [32].

## Results

**Figure 1.**
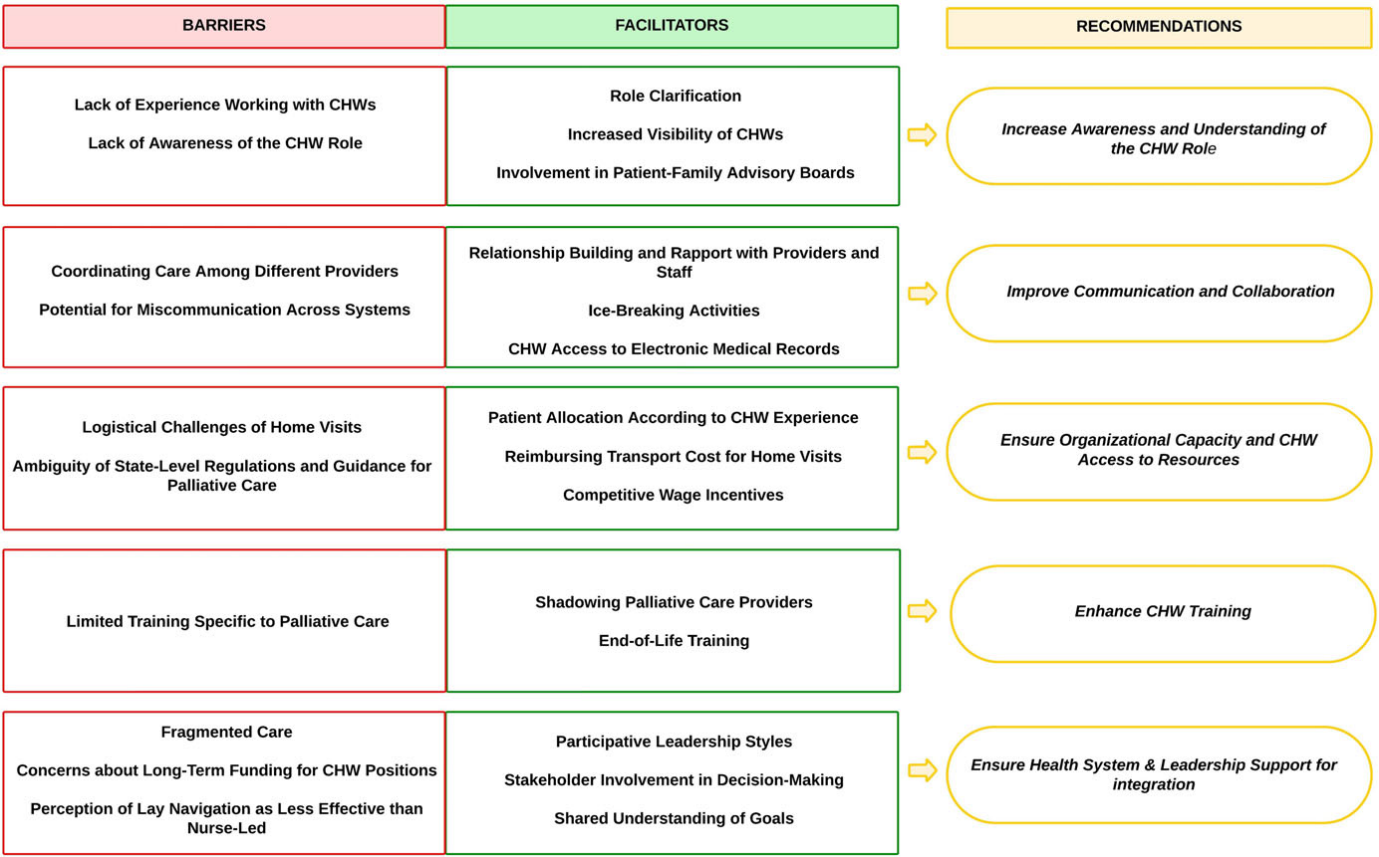
**Results summary.** CHW = community health worker.

Our sample was comprised of 25 key informants, including 6 palliative care providers, 6 oncologists, 5 cancer center leaders, 2 cancer care navigators, and 6 CHWs. The key informant’s recommendations for CHW integration clustered into five main domains, including: (1) increasing awareness and understanding of the CHW role, (2) improving communication and collaboration, (3) ensuring access to resources, (4) enhancing CHW training, and (5) ensuring leadership support for integration. Within each domain, we highlighted the perceived barriers, facilitators, and recommendations made by key informants based on their experiences (See Fig. 1).

### Increasing awareness and understanding of the CHW role

A prominent barrier cited by key informants was a lack of awareness about the CHW role. Clinicians recognized that CHWs could be helpful but identified a need for role clarity and improved education about CHWs’ value. Clearly defining roles among team members was considered important to prevent role confusion and foster trust. Clinicians highlighted the need for intentional awareness efforts at the provider level to reduce ambiguity, particularly regarding the perceived overlap of roles between CHWs and social workers. Participants expressed the need for a well-defined workflow, with each member having a clear purpose and a mechanism for contact in place. A palliative care provider highlighted the importance of education to help clinics integrate CHWs into practice*: “It’s all-around education, just awareness that that role exists, and then how best to utilize that role.” (Palliative Care Provider, KI05)*

Similarly, CHWs reported that role ambiguity led to resistance from clinic staff, which had to be overcome through time and effort. One CHW shared their personal experience overcoming such barriers in the past:

*It wasn’t easy at the beginning. There were some barriers that had to be removed. But once that clinic staff saw the value that we were adding and taking some of the burden off them as well as with – I’m going to be honest with you, as well as the social workers, that was some barriers there. We had to prove ourselves, that we’re here to help you help us and to help the patient. It’s all about the 28 patients. But the main thing going back to your question is it’s very important to have a relationship, a very good working relationship. (CHW, KI05)*

Cancer center leaders recommended increasing the visibility of CHWs in patient-facing areas of the clinic to raise awareness of their role among patients and their families. One cancer center leader suggested implementing best practice alerts, whereby both the patient and care provider could be notified by the health system when a patient with a certain diagnosis may benefit from CHW support:

*Whether it’s best practice alerts, or questionnaires… those kinds of things that would result in some sort of introduction. Those kinds of things might be helpful to get [CHWs] involved and engaged and have some awareness, such as an alert to the clinicians, alert to the cancer patients… Imagine if you had a diagnosis/visit combo that says, “Have you met one of these folks yet?” And you answer no, then it makes an introduction. (Cancer Center Leader, KI03)*

Informants also recommended leveraging patient-family advisory boards and featuring advertisements in hospital lobbies as ways to promote CHW recognition.

### Improving communication and collaboration

In the context of care coordination, clinicians voiced reluctance to trust CHWs to relay medical advice. They expressed concerns about the CHWs’ lack of clinical training and the potential for miscommunication when conveying medical information:

*I would say that you can’t use a community health worker [to relay medical advice]. That’s going to sound terrible, but honestly, I think it would have to be nurse driven if you want physicians to be talking to them [CHWs]. It’s going to be really hard to explain something to somebody who’s non-clinical, to then have them have to go explain it – like, it’s the game of telephone at that point. When it comes to pain and symptom management, I think 100%, I mean – I think they can make a phone call and say “Hey, the patient is in pain. What can we do about it?” But I would be hesitant to give anybody but a clinician any kind of advice because I would be worried that it would be a liability, misconstrued. (Palliative Care Provider, KI07)*

However, clinicians were willing to task-shift by having CHWs disseminate health information or assist with recording advance directives. They expressed that CHWs could play a crucial role in providing informational handouts and promoting health literacy. Clinicians were also willing to have CHWs reinforce important information related to disease and treatment characteristics:

*I also think providing handouts and health literacy aspects. As much as we explain it, having someone that can reiterate things and really help with understanding treatment toxicities and maybe provide some great handouts, all those things can be better because we do not have tons of time to explain lots of complicated things to patients, so if we start the conversation and then they can kind of close and finish them on it would be ideal. (Oncology Provider, KI02)*

Furthermore, key informants underscored the importance of effective communication and a collaborative approach to integration. Relationship building was a factor cited by CHWs and oncology team members as key for effective integration. It was reinforced within the context of palliative care, as a tool that would be helpful for the whole team. Some benefits described included building trust and openness within the team, which would enable problem areas to be shared and discussed. Relationship-building strategies suggested by informants included introducing CHWs at staff and faculty meetings, team-building exercises, and incorporating didactic training on relationship building and conflict resolution. One CHW stated that showing their appreciation to the clinical team through kind gestures may go a long way in building rapport and fostering strong team dynamics:

*It may mean taking some donuts, especially around the holiday time. Giving a thank you card. Look, we appreciate you all working with us. And once they see the value that you’re adding they’re going to be on board. But it’s very important to have a relationship with the physicians, with the nurses, with the front desk. Now, that front desk is the main connection there. If we just can walk in and say, look, I brought you something for lunch today. Is so-and-so here today? We need to see her. What time can we see her? Can you connect us? You’ve got to have a relationship starting with that front desk, it’s very important. (CHW, KI05)*

Access to electronic medical records, even with simple read and write privileges, was described as an important and necessary step to integrating CHWs into clinical teams. According to informants, it would allow clinicians to read the after-visit summary and draw attention to the important insights brought forth by a CHW’s home visit. Similarly, having access to medical records would allow CHWs to gain insight into patients’ needs before meeting them at their homes and may help patients view CHWs as an extension of their care team:

*As long as you have an electronic medical record, like even if you only give the CHWs read and write [privileges], you can read the after-visit summary. You can message the care team once you leave the patient’s house, or after you get finished talking to the patient. I think that is very crucial with being integrated from the community to the clinicians. When I was working at my last organization, that’s what we did. We had access to [the site’s EMR,] Epic, so we were able to read their chart before we even go out and meet the patient. You know, an overview of what we would be walking into. We won’t be blind-sided. (CHW, KI01)*

### Ensuring organizational capacity and CHWs’ access to resources

At the clinician level, informants felt that a lack of practical considerations might hinder the integration of CHWs within clinical settings. Clinicians highlighted coordination challenges between team members and were concerned that adding a member with a new role may make it more confusing and harder to regulate care. One participant shared:

*We struggle already to communicate with caregivers and family. Is this person [new CHW] physically there? How does this person communicate? I think those are the biggest sort of questions that I have – I mean, there’s no way that they can be there 24/7 with one patient. And so, like, what does that time look like? Our health systems are not set up that way, to say it’s patient navigator hour. We’re going to go around and talk to all the navigators. We have to think about that, otherwise now it’s just another person that we’re trying to chase down. (Palliative Care Provider, KI03)*

At the policy level, key informants also expressed concerns over the lack of standardized practice and ambiguity of guidelines for implementing palliative care in the inpatient and outpatient settings. Before CHWs can be fully integrated, they emphasized the need for a clear delineation of what services are covered under palliative care in the state’s statute, in terms of primary and specialty palliative care and a roadmap for implementation:

*Reimbursement is certainly [a challenge] on both hospital patients and ambulatory but more so on the ambulatory side. You know, large hospital systems can kind of absorb overhead costs and provide the service… The [state] statute talks about providing palliative care, but it doesn’t describe how to do that at the hospital level, just that it must be provided. It could be a single provider, while we have five, six, or more on the team. So, standardization is another concern across hospitalized patients. And then it’s the wild west on the ambulatory side. There are really no statutes, there are really no regulations, and there are no consumer guidelines for what it should look like, whether a single provider is a palliative care program. Is it primary palliative care, or is it advanced practice? (Cancer Center Leader, KI03)*

At the CHW level, informants drew attention to issues with patient allocation, logistics of CHW home visits, and resource allocation. One CHW informant recommended a policy of two patients at a time for newer CHWs and having a no-limit policy for more experienced CHWs. Informants also recommended providing CHWs with necessary equipment and transportation, such as work phones and laptops. One CHW shared their experience working with an organization that covered their transportation costs by reimbursing tracked mileage and fuel costs, which was especially important considering the extensive areas they covered. Many CHWs emphasized the importance of offering competitive wage incentives and opportunities for training. Participants underscored the need for hiring processes to select individuals who are truly passionate about the work. As one participant expressed:

*Me personally, I do believe in compensation because you’re going to get better results, but the main focus cannot be compensation. To be a health advisor or an advocate for someone else, you’ve got to also have a passion, it must come from within. As a patient navigator and out there throughout and traveling, we would hear so many people, “We want to do what you’re doing. Can we do what you’re doing?” It takes a special type of person to be a navigator. (CHW, KI05)*

Recommendations for having CHWs specialize in cancer-specific teams were also shared. According to one participant, this would improve practice and help patients know, “exactly who to go to.”

### Enhancing CHW training

One prominent challenge highlighted was a perceived lack of standardized discipline-specific training programs for CHWs. Informants emphasized that didactic training specific to health diagnoses should be integral for integration. One informant shared how an understanding of what lowered immunity entailed for a cancer patient and knowledge of potential treatments would help CHWs be effective patient advocates and care team members. Furthermore, informants felt that observational training, such as shadowing providers in the clinical setting, would be important. One provider shared how shadowing could help CHWs better understand the medical aspects of care and adjust to the provider work style:

*I think it would be helpful for them to almost shadow certain providers just to see what the different aspects really look like. Each individual provider can be a little different*. (*Oncologist, KI02)*

Informants also emphasized that the current healthcare systems lack practical training in palliative care for CHWs, which would be an important antecedent to their integration, especially for the development of skills specific to palliative care, including end-of-life training. CHWs also shared that forming close connections with individuals can be emotionally challenging; therefore, they emphasized that training should enable them to address the emotional needs of caregivers and family members. The ability to interpret body language and address concerns without judgment were considered important:

*“I think you must absolutely cover getting comfortable with having hard conversations because that is certainly a big part of it. I’ll be honest, I feel like one of my biggest advantages in doing this job is if I went in a room of 20 family members, being able, if you will, to read the room. You’ve got to look around. I would watch people’s expressions, their body language. So, somebody needs to be really aware of that and pay attention because I really could tell who was on board and who wasn’t or who had questions or who wasn’t getting it at all and without calling them out, saying “Well, you know, this could really be confusing. So, let me explain it this way,” or “A lot of people feel this way about this. However…” So that you’re kind of answering. (CHW, KI06)*

### Ensure health system and leadership support for integration

Informants in a leadership role highlighted concerns for care fragmentation and cost coverage for CHW-delivered services. Informants described how CHW compensation is typically provided through research grants and shared that sustainable funding mechanisms for CHWs in the USA remain limited. An informant comparing the CHW integration in Brazil to that in the USA stated:

*Community health workers here [in the US]… I work with that model, have worked my entire career, is we employ them for the research project, and then when it’s done, they lose their jobs, and then the healthcare system never absorbs those individuals. There’s some movement here in the US now to do so. The discussion is who’s going to pay for it. The healthcare system does not want to pay for it and the patient cannot absorb that cost. So, then what? (Cancer Center Leader, KI08)*

A few informants also expressed concern that providers may perceive lay navigation as less potentially successful than nurse-led navigation, which may influence stakeholder buy-in for CHW integration. One leader shared their experience of implementing a nurse-led navigation initiative:

*We have navigators in a couple of our locations. They’re actually transitioning out of what we’re calling navigators to calling them office practice nurses… they really are tasked with all the things that you described, helping people enter and move through the system and make those care transitions. Being nursing-led has been tremendously helpful in comparison to things like navigator roles that are more social work based or lay-person based, which focus on some other things. Navigation is not widely used in my observation at [institution] by any of the definitions that we’ve talked about. I’m not sure why that is. I think there’s been hesitancy to it in the past, because it seems to add cost to the system. I think the cynical view of navigation is that it’s a role that people create when their processes stink. I do not know that that’s fully fair, but it’s also not completely untrue. (Cancer Center Leader, KI02)*

The interviews highlighted the strong need for ensuring leadership support. When discussing the implementation of a new healthcare strategy, informants emphasized leadership styles centered on stakeholder involvement. Drawing from experiences, they recommended that new leaders acquire a contextual understanding of the problem and involve key stakeholders during decision-making in a genuine, purposeful way. As one informant stated:

*You get all stakeholders in a room, let them have a stake in the game, and work towards a common goal. That’s the most effective way for major kinds of change and minor kinds of change – have full engagement, full transparency, and everybody’s got skin in the game. (Cancer Center Leader, KI03)*

A cancer center leader with 25 years of experience in the health sector described the implementation of an intervention whereby success was intricately linked to change management, team-building efforts, physician leadership engaging frontline staff, along with clear communication of goals and the “why” behind the implementation of the intervention:

*We’re taking from that first experience a lot of lessons that we’ve learned and applying it to the others when we’re getting ready to go forward. Honestly, it had to do a lot with change management more than anything. It was about team building. It was about people understanding the “why” behind the changes that we were making. (Cancer Center Leader, KI02)*

## Discussion

Our study describes five main domains of recommendations surrounding facilitators of and barriers to CHW integration within palliative care teams as perceived by key informants. Key informants principally recommended increasing CHW role awareness, improving communication and collaboration between team members – with a special focus on relationship building – and providing training opportunities relevant to palliative care. There was a strong emphasis on incentivizing engagement through the provision of necessary equipment and compensation for effort, as well as ensuring leadership support. Informants highlighted the need for sustainable reimbursement models to support CHW integration over the long term. Our findings build on the existing literature on the role of CHWs within specialty care practice and provide a framework for further research into their integration within palliative care.

CHWs have served in many different capacities as members of the healthcare workforce, particularly in under-resourced populations [33]. This heterogeneity in role adds to role confusion especially when integrating CHWs within a new system such as specialty care practice in the USA. Clinical staff’s lack of understanding of the CHW’s role is a common finding across the literature. Providers do not always understand what CHWs are achieving and therefore struggle to meaningfully engage with them [34–36]. Optimal integration entails clear assignment of roles between team members and recognition of all roles as valuable. CHWs’ unique ability to go into patients’ homes to optimize nonmedical aspects of care helps differentiate them from other care team members, such as social workers or nurse navigators. Transparency in the CHW hiring process, with a clearly outlined job description, selection metrics, and evaluation criteria can help further establish role efficacy and distinguish the CHW role [37]. Moreover, CHWs often must advocate for themselves. CHW participants reported facing resistance from clinical staff and having to gradually earn the trust of team members over time. While trust-building is important, ensuring the education of the care team on how to effectively utilize a CHW’s skillset can prevent CHWs from being marginalized in clinical settings [38,39].

Health systems can incentivize CHW integration through comprehensive training opportunities and providing them with access to resources. CHWs are most effective when they are given access to health records and included in care decisions within multidisciplinary teams [39]. Our CHW participants emphasized how access to health records helped them better understand their patients and provide a feedback loop to physicians on patients’ care needs. Furthermore, although training in interpersonal skills and managerial tasks, such as relationship building and resource navigation is common, few programs have been reported to cover training specific to orientating CHWs with the new role [37]. There is also a lack of standardization of the training methodology for CHWs across institutions, in contrast to training processes for other roles in the healthcare workforce. In the context of palliative care, in addition to didactic training on diagnoses and treatment plans, informants emphasized role-specific training, such as conversational skills required in dealing with palliative care patients and their families, as well as training on effectively coordinating care with clinical team members. Finally, our recommendations echoed similar calls for sustainable reimbursement structures to support the long-term integration of CHWs into health systems [35,38]. CHW-integrated models have been shown to be cost-effective through generating increased return on investments and lowering annual expenditure for patients [38–41]. However, as expressed by our informants and shown in the literature, most funding for CHWs in the USA comes through research grants [42]. Encouragingly, public insurance coverage of some CHW services is available through Medicaid and Medicare [43,44]. In addition to funding, the organizational environment needs to be receptive to the incorporation of CHW positions. Our findings highlight the importance of documentation of CHW efforts and outcomes and leadership’s willingness to endorse CHW engagement. Informants also underscored the importance of viewing CHW integration within the context of existing disparities and engaging key stakeholders in decision-making to facilitate integration. Although these recommendations need further study to determine the full extent of their impact on facilitating CHW integration, our findings suggest that for health systems to fully incorporate the CHW role, long-standing efforts at role clarification, engagement, and sustainable reimbursement models are essential.

## Conclusion

To address disparities in palliative care access, we explored key informants’ recommendations for CHW integration within palliative care teams. These included increasing role awareness and provision of role-specific training at the provider and CHW levels and making practical and logistical adjustments at the institutional level. Promoting CHW engagement through involvement in care team discussions, access to electronic health records, and advisory board participation can facilitate their integration. Recommendations for policymakers include pushing for sustainable reimbursement models so the full potential of CHW integration into specialty care can be realized long term. Our recommendations serve as signposts for further empirical research in CHW integration within palliative care.

## References

[1] Smith CB , Phillips T , Smith TJ. Using the new ASCO clinical practice guideline for palliative care concurrent with oncology care using the TEAM approach. Am Soc Clin Oncol Educ Book. 2017;37:714–723. 28561696 10.1200/EDBK_175474

[2] Bazargan M , Bazargan-Hejazi S. Disparities in palliative and hospice care and completion of advance care planning and directives among non-Hispanic blacks: a scoping review of recent literature. Am J Hosp Palliat Med. 2021;38(6):688–718. 10.1177/1049909120966585 PMC8083078 33287561

[3] Lee KT , George M , Lowry S , Ashing KT. A review and considerations on palliative care improvements for African Americans with cancer. Am J Hosp Palliat Med. 2021;38(6):671–677. 10.1177/1049909120930205 32489113

[4] Boucher NA , Raghavan M , Smith A , Arnold R , Johnson KS. Palliative care in the African American community #204. J Palliat Med. 2016;19(2):228–230. 26840858 10.1089/jpm.2015.0523

[5] Improving Palliative Care for Cancer [Internet].Washington, D.C.: National Academies Press, 2001. [cited 2024 Mar 4]. Available from: http://www.nap.edu/catalog/10149.

[6] Rhodes RL , Batchelor K , Lee SC , Halm EA. Barriers to end-of-life care for African Americans from the providers’ perspective: opportunity for intervention development. Am J Hosp Palliat Med. 2015;32(2):137–143. 10.1177/1049909113507127 PMC5508735 24097838

[7] Carr D. Racial differences in end-of-life planning: why don’t Blacks and Latinos prepare for the inevitable? OMEGA. J Death Dying. 2011;63(1):1–20. 10.2190/OM.63.1.a 21748919

[8] Johnson KS , Elbert-Avila KI , Tulsky JA. The influence of spiritual beliefs and practices on the treatment preferences of African Americans: a review of the literature. J Am Geriatr Soc. 2005;53(4):711–719. 15817022 10.1111/j.1532-5415.2005.53224.x

[9] Braun UK , Rabeneck L , McCullough LB , et al. Decreasing use of percutaneous endoscopic gastrostomy tube feeding for veterans with dementia—Racial differences remain. J Am Geriatr Soc. 2005;53(2):242–248. 15673347 10.1111/j.1532-5415.2005.53109.x

[10] Zogby CB. Burnout among palliative care providers. J Am Assoc Nurse Pract. 2023;35(11):676–681. 37395681 10.1097/JXX.0000000000000912

[11] Fasolino T , Mayfield ME , Valentine K , Rosa WE , Koci A. Palliative care in rural communities. AJN Am J Nurs. 2024;124(8):50–55. 10.1097/01.NAJ.0001027716.70431.35 PMC11616013 39051815

[12] Evans BA , Turner MC , Gloria JN , Pickett LC , Galanos AN. Palliative care consultation is underutilized in critically ill general surgery patients. Am J Hosp Palliat Med. 2020;37(2):149–153. 10.1177/1049909119864025 31315425

[13] Flieger SP , Chui K , Koch-Weser S. Lack of awareness and common misconceptions about palliative care among adults: insights from a national survey. J Gen Intern Med. 2020;35(7):2059–2064. 32157652 10.1007/s11606-020-05730-4 PMC7351936

[14] Johnston FM , Neiman JH , Parmley LE , et al. Stakeholder perspectives on the use of community health workers to improve palliative care use by African Americans with cancer. J Palliat Med. 2019;22(3):302–306. 30388060 10.1089/jpm.2018.0366 PMC6391609

[15] American Public Health Association (APHA) (n.d.). Community health workers. (https://www.apha.org/apha-communities/member-sections/community-health-workers) Accessed April 3, 2024. 10.2190/NS.20.3.l 20943481

[16] National Center for Chronic Disease Prevention and Health Promotion (U.S.). Division for Heart Disease and Stroke Prevention. Addressing chronic disease through community health workers : a policy and systems-level approach : a policy brief on community health workers. 2nd edition. April 2015. URL: https://stacks.cdc.gov/view/cdc/30816.

[17] Van Iseghem T , Jacobs I , Vanden Bossche D , et al. The role of community health workers in primary healthcare in the WHO-EU region: a scoping review. Int J Equity Health. 2023;22(1):134. 37474937 10.1186/s12939-023-01944-0 PMC10357780

[18] U.S. Department of Homeland Security Cybersecurity and Infrastructure Security Agency, Krebs C. *Memorandum on identification of essential critical infrastructure workers during COVID-19 response*. 2020.

[19] Boyce MR , Katz R. Community health workers and pandemic preparedness: current and prospective roles. Front Public Health. 2019;7:62. 30972316 10.3389/fpubh.2019.00062 PMC6443984

[20] Gary TL , Bone LR , Hill MN , et al. Randomized controlled trial of the effects of nurse case manager and community health worker interventions on risk factors for diabetes-related complications in urban African Americans. Prev Med. 2003;37(1):23–32. 12799126 10.1016/s0091-7435(03)00040-9

[21] Brownstein JN , Bone LR , Dennison CR , Hill MN , Kim MT , Levine DM. Community health workers as interventionists in the prevention and control of heart disease and stroke. Am J Prev Med. 2005;29(5):128–133. 16389138 10.1016/j.amepre.2005.07.024

[22] Brownstein JN , Chowdhury FM , Norris SL , et al. Effectiveness of community health workers in the care of people with hypertension. Am J Prev Med. 2007;32(5):435–447. 17478270 10.1016/j.amepre.2007.01.011

[23] Hand T , Rosseau NA , Stiles CE , et al. The global role, impact, and limitations of community health workers (CHWs) in breast cancer screening: a scoping review and recommendations to promote health equity for all. Glob Health Action. 2021;14(1):1883336. 33899695 10.1080/16549716.2021.1883336 PMC8079044

[24] Garcia ML , Sprager L , Jiménez EB. Latino community health workers: meeting their community’s emotional needs in intuitively culturally appropriate ways. Prog Community Health Partnersh Res Educ Action. 2022;16(1):17–25. 10.1353/cpr.2022.0002 35342108

[25] Perales J , Reininger BM , Lee M , Linder SH. Participants’ perceptions of interactions with community health workers who promote behavior change: a qualitative characterization from participants with normal, depressive and anxious mood states. Int J Equity Health. 2018;17(1):19. 29402278 10.1186/s12939-018-0729-9 PMC5800056

[26] Knowles M , Crowley AP , Vasan A , Kangovi S. Community health worker integration with and effectiveness in health care and public health in the United States. Annu Rev Public Health. 2023;44(1):363–381. 37010928 10.1146/annurev-publhealth-071521-031648

[27] Siddiqi A , Monton O , Woods A , et al. Dissemination and implementation of a community health worker intervention for disparities in palliative care (DeCIDE PC): a study protocol for a hybrid type 1 randomized controlled trial. BMC Palliat Care. 2023;22(1):139. 37718442 10.1186/s12904-023-01250-0 PMC10506196

[28] Woods AP , Monton O , Fuller SM , et al. Implementation barriers and recommendations for a multisite community health worker intervention in palliative care for African American oncology patients: a qualitative study. J Palliat Med. 2024;27(9):1125–1134. 38716800 10.1089/jpm.2023.0703 PMC12372906

[29] Damschroder LJ , Reardon CM , Widerquist MAO , Lowery J. The updated consolidated framework for implementation research based on user feedback. Implement Sci. 2022;17(1):75. 36309746 10.1186/s13012-022-01245-0 PMC9617234

[30] Damschroder LJ , Aron DC , Keith RE , Kirsh SR , Alexander JA , Lowery JC. Fostering implementation of health services research findings into practice: a consolidated framework for advancing implementation science. Implement Sci. 2009;4(1):50. 19664226 10.1186/1748-5908-4-50 PMC2736161

[31] Glasgow RE , Vogt TM , Boles SM. Evaluating the public health impact of health promotion interventions: the RE-AIM framework. Am J Public Health. 1999;89(9):1322–1327. 10474547 10.2105/ajph.89.9.1322 PMC1508772

[32] Tong A , Sainsbury P , Craig J. Consolidated criteria for reporting qualitative research (COREQ): a 32-item checklist for interviews and focus groups. Int J Qual Health Care. 2007;19(6):349–357. 17872937 10.1093/intqhc/mzm042

[33] Washington DC:USDHHS, Health Resources and Services Administration. *USDHHS Community Health Worker National Workforce Study*. 2007.

[34] Minore B , Boone M. Realizing potential: improving interdisciplinary professional/paraprofessional health care teams in Canada’s northern aboriginal communities through education. J Interprof Care. 2002;16(2):139–147. 12028894 10.1080/13561820220124157

[35] Payne J , Razi S , Emery K , Quattrone W , Tardif-Douglin M. Integrating community health workers (CHWs) into health care organizations. J Community Health. 2017;42(5):983–990. 28391593 10.1007/s10900-017-0345-4

[36] Smithwick J , Nance J , Covington-Kolb S , Rodriguez A , Young M. Community health workers bring value and deserve to be valued too:, key considerations in improving CHW career advancement opportunities. Front Public Health. 2023;11:1036481. 36969656 10.3389/fpubh.2023.1036481 PMC10030954

[37] O’Brien MJ , Squires AP , Bixby RA , Larson SC. Role development of community health workers. Am J Prev Med. 2009;37(6):S262–S269. 19896028 10.1016/j.amepre.2009.08.011 PMC2856599

[38] Pittman M , Sunderland A , Broderick A , Barnett K. Bringing Community Health Workers into the Mainstream of U.S. Health Care, NAM Perspect [Internet], 2015 Feb 4 [cited 2024 Mar 4], 5(2). Available from:https://nam.edu/perspectives-2015-bringing-community-health-workers-into-the-mainstream-of-u-s-health-care-2/.

[39] Hostetter Martha , Klein Sarah. In focus: integrating community health workers into care teams. *The Commonwealth Fund*. 2015

[40] Stupplebeen DA , Sentell TL , Pirkle CM , et al. Community health workers in action: community-clinical linkages for diabetes prevention and hypertension management at 3 community health centers. Hawaii J Med Public Health J Asia Pac Med Public Health. 2019;78(6 Suppl 1):15–22. PMC6603891 31285963

[41] Pinto D , Carroll-Scott A , Christmas T , Heidig M , Turchi R. Community health workers: improving population health through integration into healthcare systems. Curr Opin Pediatr. 2020;32(5):674–682. 32889962 10.1097/MOP.0000000000000940

[42] Schmit CD , Washburn DJ , LaFleur M , Martinez D , Thompson E , Callaghan T. Community health worker sustainability: funding, payment, and reimbursement laws in the United States. Public Health Reports®. 2022;137(3):597–603. 33909522 10.1177/00333549211006072 PMC9109543

[43] Haldar S , Hinton E. State policies for expanding medicaid coverage of community health worker (CHW) services [Internet]. *Kaiser Family Foundation*, 2023, Available from: https://www.kff.org/medicaid/issue-brief/state-policies-for-expanding-medicaid-coverage-of-community-health-worker-chw-services/.

[44] Medicare Parts A & B. CMS Physician Payment Rule Advances Health Equity [Internet]. Centers for Medicare & Medicaid Services. 2023[cited 2023 Dec 4], Available from: https://www.cms.gov/newsroom/press-releases/cms-physician-payment-rule-advances-health-equity.

